# Salinity-Driven Changes in Behavioral Responses of Catadromous *Eriocher sinensis*

**DOI:** 10.3390/ani12172244

**Published:** 2022-08-30

**Authors:** Chenchen Shen, Ruifang Wang, Guangpeng Feng, Feng Zhao, Tao Zhang, Xiaorong Huang

**Affiliations:** 1 East China Sea Fisheries Research Institute, Chinese Academy of Fishery Sciences, Shanghai 200090, China; 2 College of Fisheries and Life Sciences, Shanghai Ocean University, Shanghai 200090, China; 3 College of Animal Science, Inner Mongolia Agricultural University, Hohhot 010018, China

**Keywords:** *Eriocher sinensis*, salinity fluctuationun, behavior, catadromous migration

## Abstract

**Simple Summary:**

Salinity is an important environmental factor which can influence the behavior of *Eriocheir sinensis*. In this study, female crabs were more active in a saline environment, especially low salinity stress, and the changes of antennae were obviously different under salinity shifts. Interestingly, *E. sinensis* had obvious behavioral differences in the high and low salinity stress, suggesting *E. sinensis* has different behaviors to adapt to the change of water salinity.

**Abstract:**

The effects of salinity on behavior are far-reaching, and *Eriocheir sinensis* showed disparate behaviors under different salinity conditions. Female crabs were more active in saline water, especially low salinity stress, which is beneficial for female crabs to escape from the low-salinity environment quickly. Then, antennal movement indicated that antennae might be the main osmoreceptors in *E. sinensis*, and 65 min might be a good starting time for salinity stress to analyze osmoregulation in crabs. Interestingly, *E. sinensis* had obvious behavioral differences in the high and low salinity stress, and behaviors were more intense in a salinity dip from salinity 18 to salinity 0. This study analyzed the osmoregulatory process of catadromous *E. sinensis* in different salinity from the point of osmoregulatory organ and behavioral response. These results will provide a scientific basis for the osmoregulatory mechanism of *E. sinensis*, which are conducive to evaluating and analyzing the impact of saltwater intrusion in the Yangtze River estuary on resource fluctuation.

## 1. Introduction

Chinese mitten crab, *Eriocheir sinensis*, belongs to an important catadromous crab in the Yangtze River of China [1]. The parent *E. sinensis,* finishing reproductive molting in freshwater need to migrate to the brackish estuary for reproduction in November [2]. The Yangtze River estuary is the largest and most high-quality spawning ground of *E. sinensis* in China [3]. The present estuary is located at the junction of the Yangtze River and the East China Sea, so the salinity varies greatly (salinity 0–35) which is heavily influenced by tidal action and seasons [4,5]. For parent crabs, the suitable salinity is 15–23 for mating, while salinity of 10–17 for spawning [6,7]. Freshwater (salinity < 0.5) can reduce the gonadal maturity of parent crabs and restrain the mating behavior [8,9]. The suitably increased salinity might stimulate the occurrence of reproductive activity, but parent crabs did not lay eggs at salinity 30, suggesting excessive salinity has a bad effect on crabs [6,10]. Therefore, salinity is one of the key environmental factors affecting *E. sinensis* in the Yangtze River estuary, and the adaptation to salinity changes is very important for the survival and subsequent reproduction of *E. sinensis*.

Environmental variability is common for aquatic animals, and environmental changes are always related to physiological response, which might affect growth, feeding and reproduction [11]. Facing environmental challenges, aquatic animals would make behavioral changes which have far-reaching influence for both their fitness and survival [12]. For example, *Pagurus bernhardus* in low pH significantly reduced antennular flick rates (the ‘sniffing’ response in decapod) and movement [13]. In the increased temperature, *Apistogramma agassizii* and *Mesonauta insignis* increased both activity level and digging behavior and decreased the use of shelter [14]. Additionally, the salinity in water is always changing due to tidal action and seasonal variation, forcing aquatic animals to start osmotic regulation to reach a new equilibrium [15,16]. Previous reports have confirmed that the morphology, behavior and physiology of aquatic animals, such as feeding, movement, defense and fighting behavior changed under salinity stress,. For instance, *Macrobrachium rosenbergii* increased searching time and decreased feeding and exercising time at relatively high salinity, directly leading to decreased food intake [17]. The fighting behavior (frequency and time) of *Procambarus clarkii* at low salinity was significantly stronger than that at high salinity [18]. Crabs can regulate a variety of behaviors to control osmosis for survival in a wide range of salinity [19]. *Callinectes sapidus* and *Carcinus maenas*, which were classed as efficient osmoregulators, increased these behaviors (mouthpart movement, abdomen extension, antennules flick and cleaning of antennae) with decreasing salinity; *Cancer magister* and *Libinia emarginata*, weak osmoregulators, exhibited lesser aforementioned behaviors with decreasing salinity and became inactive at lower salinity [20].

For *E. sinensis*, a number of studies on the adaptation to salinity have focused on physiological changes and molecular mechanisms [21,22]. For example, the activity of digestive enzymes reduced with the elevated salinities from 0 to 35, indicating that the digestive capacity of *E. sinensis* became weak, and female crabs were more tolerant of high salinity than male crabs from the perspective of digestive modulation [23]. Mature *E. sinensis* can decrease Na^+^/K^+^-ATPase and carbonic anhydrase activity at increasing salinities to reduce Na^+^ intake [24]. Meanwhile, the expression of genes related to osmoregulation, such as Na^+^/K^+^- ATPase, H^+^-ATPase and glutathione S-transferase, were significantly changed under salinity stress, and the differentially expressed genes were also found in different tissues [25,26,27]. However, the behavioral changes in catadromous *E. sinensis* after salinity stress are ignored. *E. sinensis*, which had extreme eurysalinity and a strong osmoregulation ability to maintain homeostasis under salinity stress, was often used as the model species for studying the osmoregulation of crustaceans in different salinity conditions, but the research about osmoreceptor is scarce [28,29,30].

Thus, it is significant to analyze the osmoregulatory processes of catadromous *E. sinensis* which were subjected to changes of environmental salinity from the point of osmoregulatory organ and behavioral responses. This study will promote the understanding of osmoregulation and organ function in crustaceans, then evaluate and analyze the impact of saltwater intrusion in the Yangtze River estuary on resource fluctuation.

## 2. Materials and Methods

### 2.1. Animal Collection and Laboratory Setup

**Figure 1:**
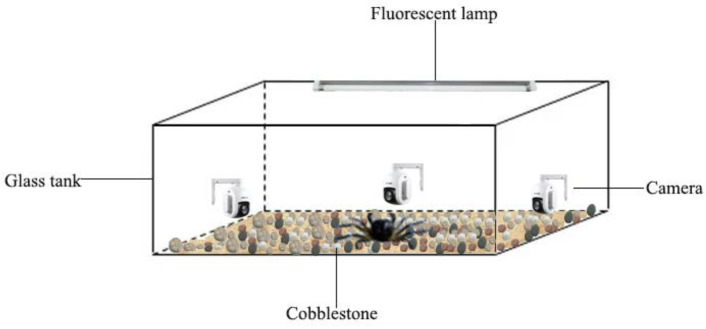
The experiment device of behavior observation.

Adult female *E. sinensis* were collected from Baxu Wharf in Jiangyin, P.R. China (120°27′ S, 31°95′ W, salinity < 0.5, temperature 19 °C) in December. The average weight of female crabs was 102.03 ± 7.49 g, and the average shell width was 62.18 ± 11.63 mm. During a week of acclimation, female crabs were fed with fresh snail meat at the 1% of the crab weight every three days. The experimental water quality parameters were monitored daily to maintain conditions of temperature 18 ± 1 °C, dissolved oxygen > 6 mg/L, pH 7.0 ± 0.5, salinity < 0.5, total ammonia nitrogen < 0.2 mg/L and a natural light/dark photoperiod (14 L:10 D). The depth of water was kept above 15 cm. The two-thirds of water in each tank was replaced every other day and the bottom dirt cleaned daily. Then, female crabs with healthy, complete appendages and consistent specifications were selected for the behavioral experiment. The experiment device was a transparent aquarium (40 × 25 × 30 cm). A layer of sand and pebbles (diameter 2 cm) were laid at the bottom of the aquarium as shelter to facilitate crabs crawling (Figure 1). Behavioral observation was carried out in a closed laboratory, and a 20 W daylight lamp was installed above the aquarium to ensure the stability of the light source. The automatic cameras with real time monitoring were installed on the side of the aquarium for subsequent computer video analysis.

### 2.2. Behavioral Experiment

Four treatment groups were set up: freshwater control group (FC) which was salinity 0; salinity control group (SC) which was salinity 18; salinity surge group (SS) which surged from salinity 18 to salinity 30 and salinity dip group (SD) which dipped from salinity 18 to salinity 0. In the SC group, the salinity was increased by salinity 3 per day from freshwater to salinity 18 and maintained at salinity 18 for 3 days. In the experiment of FC and SC groups, the crabs (individual as a unit) were gently removed and put into aquariums for behavior observation, and the behaviors of the crabs were recorded after an hour of adaptation. In the experiment of SS and SD groups, aquariums were filled with brackish water with salinity 18 in advance, and the crabs (individual as a unit) were gently removed and put into the aquarium. After an hour of adaptation, the solution of salinity 18 was sucked out by a soft siphon, and the test solution of salinity 30 and freshwater with the same volume and temperature were added into aquariums, respectively. The salinity change was completed within 5 min to minimize the interference of changing the test solution to crabs. The behaviors of crabs in the SS and SD groups were recorded after 5 min of salinity change.

The recording time of all experimental groups was set as the starting point (0 min), and the 5th, 10th, 15th, 20th, 30th, 40th, 50th, 65th, 80th, 100th, 120th, 150th and 180th minutes were taken as the starting point of subsequent recording [31]. The behaviors of crab were recorded at each time point for 1 min, and each treatment group had four replicates. After the experiment, the video was analyzed on the computer. Eight behavioral indicators (Table 1) were counted according to previously reported methods [17]. The frequency (total times) of locomotor activity, mouthpart movement, cleaning of antennae, flick of the second antennae, eyestalk movement and the time (s) of antennules retraction, abdomen extension and closure behavior were recorded, respectively. To ensure accuracy, each indicator was proofread three times.

### 2.3. Statistical Analysis

These results of all behavioral indicators in different treatment groups were the mean values of data obtained in the four replicates. One-way Analysis of Variance (ANOVA) was used to compare differences, and Tukey’s HSD method was used for multiple comparison of data. All data were analyzed by SPSS 24.0 (IBM, Armonk, NY, USA) software.

## 3. Results

### 3.1. Locomotor Activity and Mouthpart Movement

**Figure 2:**
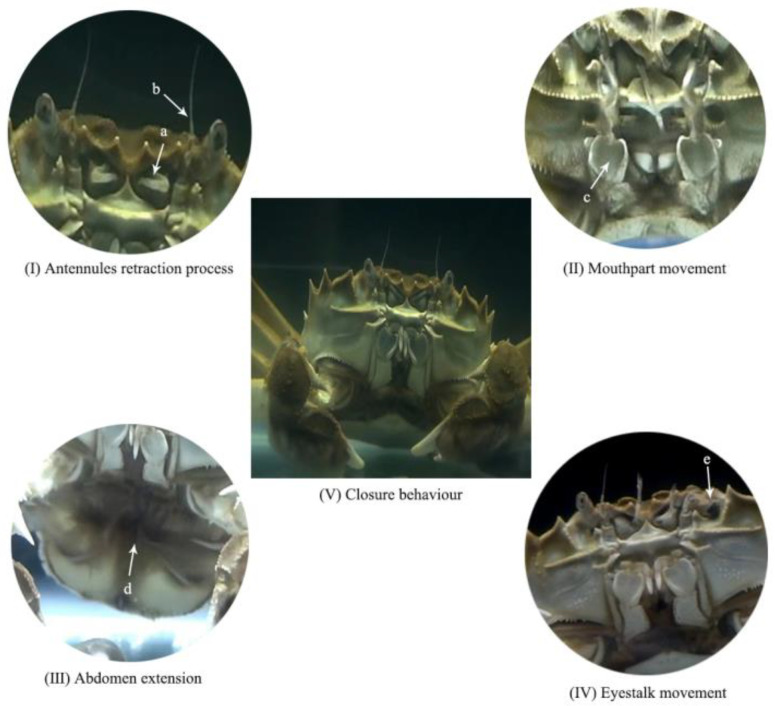
The main behaviors of *Eriocheir sinensis* in the experimental process (a: antennules; b: the second antennae; c: the third maxillipeds; d: abdomen; e: eyestalk).

**Figure 3:**
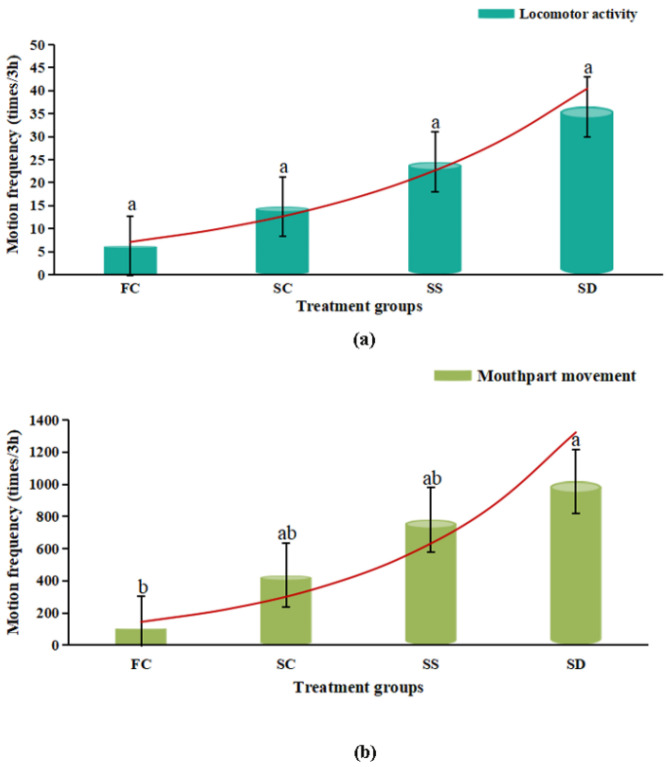
Locomotor activity (**a**); and mouthpart movement (**b**); in *Eriocheir sinensis* during 3 h exposure in four treatment groups (FC: freshwater control group; SC: salinity control group; SS: salinity surge group; SD: salinity dip group), and the red curve represents the exponential trend line. Bars with the same lowercase letters on the top indicate insignificant differences (*p* > 0.05), and bars with different letters on the top indicate significant differences (*p* < 0.05).

Locomotor activity and mouthpart movement (Figure 2II and Figure 3) showed a consistent behavioral pattern at different salinity. Compared with the FC group, the locomotor activity and mouthpart movement of crabs were enhanced in salinity groups (SC, SS and SD). Then, the locomotor activity of the SD group was the strongest, and the mouthpart movement in the SD group was significantly higher than that in the FC group (*p* < 0.05). Additionally, faced with sudden changes in salinity, the frequency of locomotor activity and mouthpart movement in crabs increased, which was the highest in the SD group.

### 3.2. Cleaning of Antennae, Flicking of the Second Antennae and Eyestalk Movement

**Figure 4:**
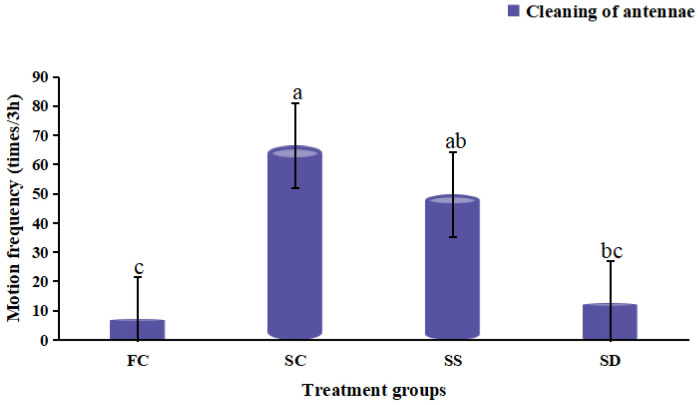
Cleaning of antennae in *Eriocheir sinensis* during 3 h exposure in four treatment groups (FC: freshwater control group; SC: salinity control group; SS: salinity surge group; SD: salinity dip group). Bars with the same lowercase letters on the top indicate insignificant differences (*p* > 0.05), and bars with different letters on the top indicate significant differences (*p* < 0.05).

**Figure 5:**
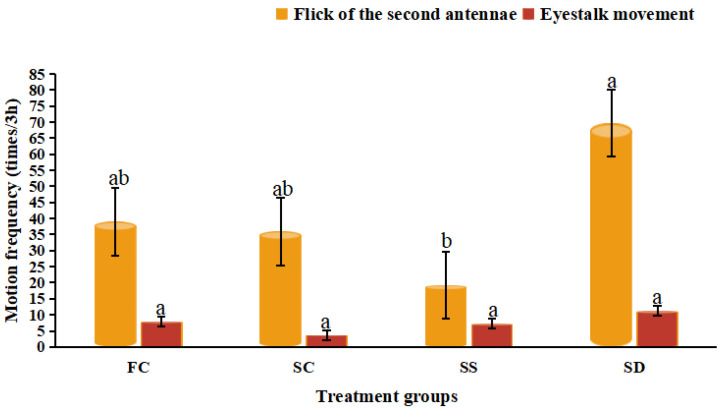
Eyestalk movement and flick of the second antennae in *Eriocheir sinensis* during 3 h exposure in four treatment groups (FC: freshwater control group; SC: salinity control group; SS: salinity surge group; SD: salinity dip group). Bars with the same lowercase letters on the top indicate insignificant differences (*p* > 0.05), and bars with different letters on the top indicate significant differences (*p* < 0.05).

The frequency of antennae cleaning was the highest in the SC group, which was significantly higher than that in the FC and the SD groups (*p* < 0.05). The sudden changes in salinity (SS and SD groups) resulted in a significant decrease in the cleaning of antennae (*p* < 0.05), and the frequency of antennae cleaning in the SD group was the lowest (Figure 4). The frequency of the second antennae flick in the SS group was the lowest, and the frequency of the second antennae flick was the highest in the SD group which was significantly higher than the SS group (*p* < 0.05) (Figure 5). The frequency of the second antennae flick in the FC and the SC groups were similar without significant difference (*p* > 0.05). Compared with the FC group, the frequency of eyestalk movement (Figure 2IV) in the SC group was decreased slightly, and the eyestalk movement after salinity changes (SS and SD groups) was increased, but no significant difference was observed (*p* > 0.05).

### 3.3. Antennules Retraction, Closure Behavior and Abdomen Extension

Antennules retraction only occurred in the FC, SC and SS groups, and the time of antennules retraction generally increased with the extension of the experiment period (Table 2). Antennules in the FC and the SC groups were retracting for a long time at the 65th min, and the retraction time could reach 42–60 s/min. Closure behavior only occurred in the SC and the SS groups, and female crabs showed more closure behavior in the SD group than in the SC group (Table 2). With the prolongation of the experiment period, the time of closure behavior generally presented an upward trend. It was observed that crabs in the SC and the SS groups often closed their mouthpart; the third maxillipeds and exopodites were closed; the antennules also retracted into the shell groove, and the body remained motionless (Figure 2V). Furthermore, closure behavior in the SC group was also maintained for a long time at the 65th min, and the closed time could reach 40–58 s/min. In this study, only the SD group had abdomen extension (Figure 2III), which appeared at the 20th min and the 180th min during the experiment period, and the duration was 60 s.

### 3.4. Overall Behavior

These results showed that locomotor activity, mouthpart movement, eyestalk movement, the flick of the second antennae and abdomen extension were the most intense in the SD group. The frequency of antennae cleaning was the highest in the SC group, which decreased significantly in the SS and the SD groups. Antennules retraction and closure behavior did not occur in the SD group, but crabs showed antennules retraction and closure behavior for a long time in the SC and SS groups. Meanwhile, it was observed that the third maxillipeds opened and closed; the second antennae vibrated when crabs changed position with occasional eyestalk movement, antennae cleaning and abdomen extension.

## 4. Discussion

### 4.1. The More Active Female Crabs in Saline Environment

Female parent crabs not only need the stimulation of saltwater to sexually mature in the Yangtze River estuary, but also need to lay eggs and migrate to areas with high salinity to facilitate embryo development [32]. Therefore, female parent crabs are more dependent and tolerant of saltwater. In this study, female crabs showed more locomotor activity at salinity 18 and salinity switch conditions. This phenomenon might be directly related to the stimulation of salinity. The changes in activity of decapoda caused by salinity stimulation has been widely reported. For example, *Cancer pagurus* and *Homarus gammarus* showed a more active behavior in high salinity and could detect and avoid areas of hypersalinity [33]. Notably, it was found that female crabs were most dynamic in the salinity dip group which dipped from salinity 18 to salinity 0. It was generally believed that increased mobility could enable aquatic animals to escape from low-salinity conditions in a short time and avoid the effects of hypotonic stress [34,35]. Due to the influence of tidal sand, runoff and rainfall, the salinity of the Yangtze River estuary varies greatly [36]. For female parent *E. sinensis*, the increase of locomotor activity under the environmental changes was beneficial to escape from the low-salinity environment quickly.

### 4.2. Osmoreceptors—Antennae

Antennae, including antennules and the second antennae, play an important role as chemoreceptors in detecting salinity changes in the environment [37]. The removal of antennules in female *Porcellana platycheles* could significantly impair the ability to discriminate osmotic concentrations in different solutions [38]. Most researchers believe that the antennae were osmoreceptors, playing an essential role in salinity detection [20,39]. In this study, antennules were retracted for a long time (42–60 s/min) at salinity 18 and in the salinity surge groups (salinity 18 to 30), but this behavior did not appear in the freshwater and salinity dip groups (salinity 18 to 0). Moreover, the second antennae flicked frequently in the salinity dip group which was significantly higher than the salinity surge group. Hence, it is speculated that antennae might be the main osmoreceptors in *E. sinensis*; antennules were correlated with hypertonic stress, and the second antennae were correlated with hypotonic stress. Additionally, a sudden surge or dip in salinity could reduce antennae cleaning, decreasing antennae sensitivity. Meanwhile, the antennules are confirmed as the main olfactory organ used to distinguish stimuli in the ingestion process of crabs [40]. A previous study showed that *E. sinensis* increased foraging behavior with increasing salinity [41], suggesting that *E. sinensis* need more time to find food at different salinities, which might be caused by the decreased antennae sensitivity.

### 4.3. Possible Time Point of Osmoregulation Initiation

Due to the effect of runoff and seasons, the salinity in Yangtze River estuary varies greatly every year. It was reported that the osmotic pressure would be 506 mmHg, which increased or decreased by about 2/3 atmospheric pressure, if the salinity of seawater increased or decreased by salinity 10 [42]. Therefore, crabs need some time to adapt to a new equilibrium after salinity change. A previous research study found that osmotic pressure of *E. sinensis* increased with time (0–6 h) at a different salinity, suggesting that *E. sinensis* could adapt quickly to changes in salinity [43]. This study found that antennules retraction and closure behavior frequently appeared (42–60 s/min) at salinity 18 and salinity 30 at the same time point (65th min), then maintained for a long time over subsequent periods, indicating the 65th min was a critical time for *E. sinensis*. At present, the time of salinity treatment was 3–7 days or more in most osmoregulation researches of crabs [44,45,46]. These results after prolonged salinity stress might overlook the key initial changes in crabs which are also crucial in the osmoregulation process. Thus, 65 min might be a good time for salinity stress to analyze osmoregulation in crabs. Moreover, the total time of behaviors mentioned above at salinity 30 was more than salinity 18, implying that high salinity has a greater effect on osmoregulation of crabs.

### 4.4. Response Behavior under High and Low Salinity Stress

Adult *E. sinensis* have strong adaptability and tolerance to salinity, and salinity stimulation is one of the important conditions for mating [47]. *E. sinensis* with mature gonads could successfully mate at salinity 8–25 [43]. However, due to the effect of tidal action, runoff, wind and river regime change, the saline intrusion in the Yangtze River estuary is serious, leading to a wide range of salinity changes from salinity 0 to 35. Then the unsuitable estuarine habitat results in a decline in biological resources [26,48,49]. Faced with salinity challenge, *E. sinensis* has to adjust its behavior for survival and reproduction. In high salinity stress (salinity 18 to 30), *E. sinensis* showed obvious behavioral changes (antennules retraction, closure response and cleaning of antennae). Notably, antennules retraction and closure response did not appear in low salinity stress, suggesting crabs might decrease ion absorption from a high-salinity environment by reducing the contact between organs and the outside environment. In low salinity stress (salinity 18 to 0), *E. sinensis* showed noticeable behavioral changes (locomotor activity, mouthpart movement, eyestalk movement, flick of the second antennae and abdomen extension). Mouthpart movement mainly includes movement of the third maxillipeds and exopodites, and each movement of the third maxillipeds corresponds to a ventilatory reversal (the water inhaled from the mouthpart flows directly back into the hind gills and is discharged by feet), which helps remove debris from the gill cavity, irrigates the posterior gill and increases the active absorption of ions [7,50]. Consistent with a previous study of McGaw et al. (1999), mouthpart movement visibly increased in low salinity stress, which is beneficial to actively absorb ions in the hypotonic environment. Additionally, *E. sinensis* steadily extended its abdomen for 60 s in low salinity stress. Crustaceans could use the hindgut to regulate osmotic pressure and ion absorption [51]. After the reduction of salinity, the tissue expanded due to the increase of water content in the tissues, and the soft tissues of hindgut and rectum were exposed to water after abdomen extension, which was beneficial to absorb ions from a low-salinity environment [52]. Therefore, abdomen extension might be a behavioral strategy for osmoregulation after salinity reduction. However, for *E. sinensis*, this behavioral strategy seems to only start after the acute salinity reduction, because crabs in freshwater did not show abdomen extension in this experiment.

## 5. Conclusions

Different salinity conditions can affect the behaviors of *E. sinensis*, and then lead to the resource variation of *E. sinensis* in the Yangtze River estuary. These results showed that female crabs had stronger activity under an abrupt drop in salinity, which might be beneficial to escape from the low-salinity environment quickly. Moreover, we speculated that antennae were the main osmoreceptors in *E. sinensis*, and 65 min might be a good time for salinity stress to analyze osmoregulation in crabs. Notably, the behaviors of *E. sinensis* were different under high and low salinity stress, which were more intense in salinity drop from salinity 18 to salinity 0. In general, *E. sinensis* can change behaviors to adapt to salinity challenges, such as fast movement, antennae activity and abdominal stretch. These behavioral changes might interfere with normal mating and reproduction in *E. sinensis*, affecting resource fluctuation in the Yangtze River estuary.

## Figures and Tables

**Table 1:** Eight behavioral indicators observed in parent *Eriocher sinensis*.

Behavior	Description
Locomotor activity	Crabs changed location horizontally or vertically.
Mouthpart movement	The third maxillipeds and exopodites opened and closed in a side-to-side motion; the palps of the maxillipeds moved independently; all exopodites of the mouth parts rapidly flicked.
Cleaning of antennae	Antennae folded down towards the maxillipeds and the palps were scraped.
Antennules retraction	Antennules made continuous rapid flicking movements and extended, which would be folded backwards into a depression in the carapace for periods of time.
Flick of the second antennae	Two second antennae flicked up and down independently.
Abdomen extension	Initially, the last abdominal segment opened and closed; then the entire abdomen opened, exposing the hindgut and rectum.
Eyestalk movement	Eyestalk moved from side to side in one minute.
Closure behavior	The body was inactive; the mouth was closed; the third maxillipeds and exopodites were inactive, the antennules retracted into the shell groove.

**Table 2:** The average time (s) of antennules retraction and closure response in *Eriocheir sinensis* during 3 h exposure in four treatment groups (FC: freshwater control group; SC: salinity control group; SS: salinity surge group; SD: salinity dip group).

Experimental Period (min)	Antennules Retraction				Closure Response			
	FC (s)	SC (s)	SS (s)	SD (s)	FC (s)	SC (s)	SS (s)	SD (s)
0	0.00 ^b^	21.25 ^a^	17.50 ^a^	0.00 ^b^	0.00 ^b^	5.00 ^a^	15.00 ^a^	0.00 ^b^
5	0.00 ^b^	18.00 ^a^	27.75 ^a^	0.00 ^b^	0.00 ^b^	4.50 ^a^	15.00 ^a^	0.00 ^b^
10	0.00 ^b^	19.00 ^a^	34.50 ^a^	0.00 ^b^	0.00 ^b^	2.50 ^a^	30.00 ^a^	0.00 ^b^
15	0.00 ^b^	30.25 ^a^	32.75 ^a^	0.00 ^b^	0.00 ^b^	14.50 ^a^	27.50 ^a^	0.00 ^b^
20	0.00 ^b^	44.50 ^a^	25.50 ^a^	0.00 ^b^	0.00 ^b^	21.00 ^a^	19.50 ^a^	0.00 ^b^
30	0.00 ^b^	34.75 ^a^	21.25 ^a^	0.00 ^b^	0.00 ^b^	4.75 ^a^	17.50 ^a^	0.00 ^b^
40	1.00 ^b^	39.25 ^a^	44.00 ^a^	0.00 ^b^	0.00 ^b^	18.00 ^a^	36.25 ^a^	0.00 ^b^
50	0.00 ^b^	49.75 ^a^	39.50 ^a^	0.00 ^b^	0.00 ^b^	24.75 ^a^	37.50 ^a^	0.00 ^b^
65	13.25 ^b^	57.50 ^a^	42.50 ^a^	0.00 ^b^	0.00 ^b^	51.50 ^a^	42.50 ^a^	0.00 ^b^
80	0.00 ^b^	42.25 ^a^	45.00 ^a^	0.00 ^b^	0.00 ^b^	20.00 ^a^	40.25 ^a^	0.00 ^b^
100	0.00 ^b^	46.50 ^a^	53.00 ^a^	0.00 ^b^	0.00 ^b^	29.00 ^a^	47.50 ^a^	0.00 ^b^
120	0.00 ^b^	54.50 ^a^	60.00 ^a^	0.00 ^b^	0.00 ^b^	49.50 ^a^	55.25 ^a^	0.00 ^b^
150	2.50 ^b^	51.25 ^a^	59.00 ^a^	0.00 ^b^	0.00 ^b^	38.00 ^a^	55.00 ^a^	0.00 ^b^
180	0.00 ^b^	48.00 ^a^	57.25 ^a^	0.00 ^b^	0.00 ^b^	39.25 ^a^	45.75 ^a^	0.00 ^b^
Total	16.75 ^b^	556.75 ^a^	559.5 ^a^	0.00 ^a^	0.00 ^b^	322.25 ^a^	484.5 ^a^	0.00 ^a^

Note: Same letters indicate the insignificant differences (*p* > 0.05), and different letters indicate the significant differences (*p* < 0.05) in four groups.

## Data Availability

The data presented in this study are available in the article. Further information is available upon request from the corresponding author.
